# Biodegradation of Gramineous Lignocellulose by *Locusta migratoria manilensis* (Orthoptera: Acridoidea)

**DOI:** 10.3389/fbioe.2022.943692

**Published:** 2022-07-19

**Authors:** Hongsen Zhang, Zhenya Li, Hongfei Zhang, Yan Li, Fengqin Wang, Hui Xie, Lijuan Su, Andong Song

**Affiliations:** ^1^ College of Life Science, Henan Agricultural University, Zhengzhou, China; ^2^ Key Laboratory of Enzyme Engineering of Agricultural Microbiology, Ministry of Agriculture, Zhengzhou, China; ^3^ College of Plant Protection, Henan Agricultural University, Zhengzhou, China

**Keywords:** locust (*Locusta migratoria manilensis*), gramineous lignocellulose, biodegradation, lignocellulolytic enzyme activity, digestive system, characterization analysis

## Abstract

Exploring an efficient and green pretreatment method is an important prerequisite for the development of biorefinery. It is well known that locusts can degrade gramineous lignocellulose efficiently. Locusts can be used as a potential resource for studying plant cell wall degradation, but there are few relative studies about locusts so far. Herein, some new discoveries were revealed about elucidating the process of biodegradation of gramineous lignocellulose in *Locusta migratoria manilensis*. The enzyme activity related to lignocellulose degradation and the content of cellulose, hemicellulose, and lignin in the different gut segments of locusts fed corn leaves were measured in this study. A series of characterization analyses were conducted on corn leaves and locust feces, which included field emission scanning electron microscopy (FE-SEM), Fourier transform infrared (FTIR) spectroscopy, X-ray diffraction pattern (XRD), and thermogravimetric (TG) analysis. These results showed that the highest activities of carboxymethyl cellulase (CMCase), filter paper cellulase (FPA), and xylanase were obtained in the foregut of locusts, which strongly indicated that the foregut was the main lignocellulose degradation segment in locusts; furthermore, the majority of nutritional components were absorbed in the midgut of locusts. The activity of CMCase was significantly higher than that of xylanase, and manganese peroxidase (MnPase) activity was lowest, which might be due to the basic nutrition of locusts being cellulose and hemicellulose and not lignin based on the results of FE-SEM, FTIR, XRD, and TG analysis. Overall, these results provided a valuable insight into lignocellulosic degradation mechanisms for understanding gramineous plant cell wall deconstruction and recalcitrance in locusts, which could be useful in the development of new enzymatic pretreatment processes mimicking the locust digestive system for the biochemical conversion of lignocellulosic biomass to fuels and chemicals.

## Introduction

The most abundant biopolymer on Earth is lignocellulose, which is recognized as a major sustainable resource for biofuels and biomaterial production (Bhatia et al., 2021). The heterogeneous and irregular arrangement of cellulose, hemicellulose, and lignin construction in the cell walls of gramineous plants and woody plants results in resistance to saccharification, which provides protection against enzymatic attack in lignocellulosic materials (Chandel et al., 2018). The degradation of cellulose to fermentable sugars (saccharification) is the most important limiting step in the biorefinery process, which relies on low-activity cellulases from bacteria and/or fungi under biorefinery conditions and are easily inhibited at present (Jiang et al., 2019). Nonetheless, several species of insects in the orders Orthoptera, Coleoptera, and Dictyoptera have now been shown to produce endogenous cellulases in the midgut or salivary glands for extracting sugars from plant cell walls and for recycling the lignocellulosic biomass in nature (Su et al., 2013; Scharf, 2020). With adequate understanding of the biological systems, especially the catalytic properties of insect enzymes, biological systems will bring more hopes for improved utilization of renewable lignocellulosic biomass.

It is well known that locusts (Orthoptera: Acridoidea) have mouthparts for chewing and always feed on gramineous grasses with cellulose contents as much as 30–50%. Studies indicated that the cellulose degradation efficiency of the locust is similar to that of the termite and beetle by comparing the cellulase activity of different insects (Oppert et al., 2010; Su et al., 2013; Su et al., 2014). A 45-kDa homolog cellulase in *Dissosteira carolina* was isolated and purified from the salivary glands and the anterior foregut (Willis et al., 2010). Endoglucanase belonging to GHF9 was cloned from a whole-body cDNA library of *Teleogryllus emma*, providing the first endoglucanase clone from an orthopteran species (Kim et al., 2008). The gene fusion of endoglucanase from *T. emma* and xylanase from *Thermomyces lanuginosus* were constructed into a fusion enzyme (EG-M-Xyn), which showed great potential in improving the enzymatic hydrolysis of lignocellulose to produce fermentable sugars (Chen et al., 2018). Some species in the Acrididae family were notorious plant feeders; therefore, research on locusts had rarely focused on specific cellulolytic systems in these species but mainly on prevention and control, biological characteristics, and species classification (Zhang et al., 2019). Elucidation of the mode of lignocellulosic digestion by the locust might provide essential information toward efficient lignocellulose degradation that would be potentially useful for the production of high-value lignocellulosic-derived products and cellulosic biofuel. Therefore, the mechanism of the gramineous plant digestion process and the related enzyme systems for lignocellulose degradation in locusts are worth studying. In addition, the degradation of gramineous plants by the locust, which has not yet been documented, could be important for establishing alternate systems to degrade the gramineous plants.

In our preliminary work, the lignocellulolytic activities in the gut fluids of about 54 insect species that belong to seven different taxonomic orders were determined, and the highest carboxymethyl cellulase (CMCase) activity in the gut fluids were found in Coleoptera and Orthoptera (Su et al., 2013; Su et al., 2014). Therefore, the purposes of this study are as follows: 1) to estimate enzyme activity assays related to lignocellulosic degradation in the gut fluids of *Locusta migratoria manilensis*; 2) to measure the content of cellulose, hemicelluloses, and lignin in corn leaves in the different gut segments of the locust; and 3) to analyze the physical–chemical properties of locust feces using field emission scanning electron microscopy (FE-SEM), Fourier transform infrared (FTIR) spectroscopy, X-ray diffraction (XRD) pattern, and thermogravimetric (TG) analysis/differential thermogravimetry (DTG). Overall, the results provide insight into lignocellulose degradation mechanisms for understanding plant cell wall deconstruction, which could be useful in the development of new biological pretreatment processes.

## Materials and Methods

### Sample Collection and Preparation of Crude Enzyme Liquid

Locust (*L. migratoria manilensis*) samples were provided by Insect Cultivation Co., Ltd., of Xingyang City in China. Locusts were supplied with *ad libitum* fresh corn leaf blades in the covering with voile (0.5 × 0.5 × 0.5 m) at room temperature after 1 week. Once they reached adulthood, they were divided into three groups (about 100 locusts per group) for three biological replicate samples. The feces and orts of adult locusts were collected every day and stored at −20°C until being freeze-dried to a constant weight. The feces and orts were separated and weighed.

**FIGURE 1:**
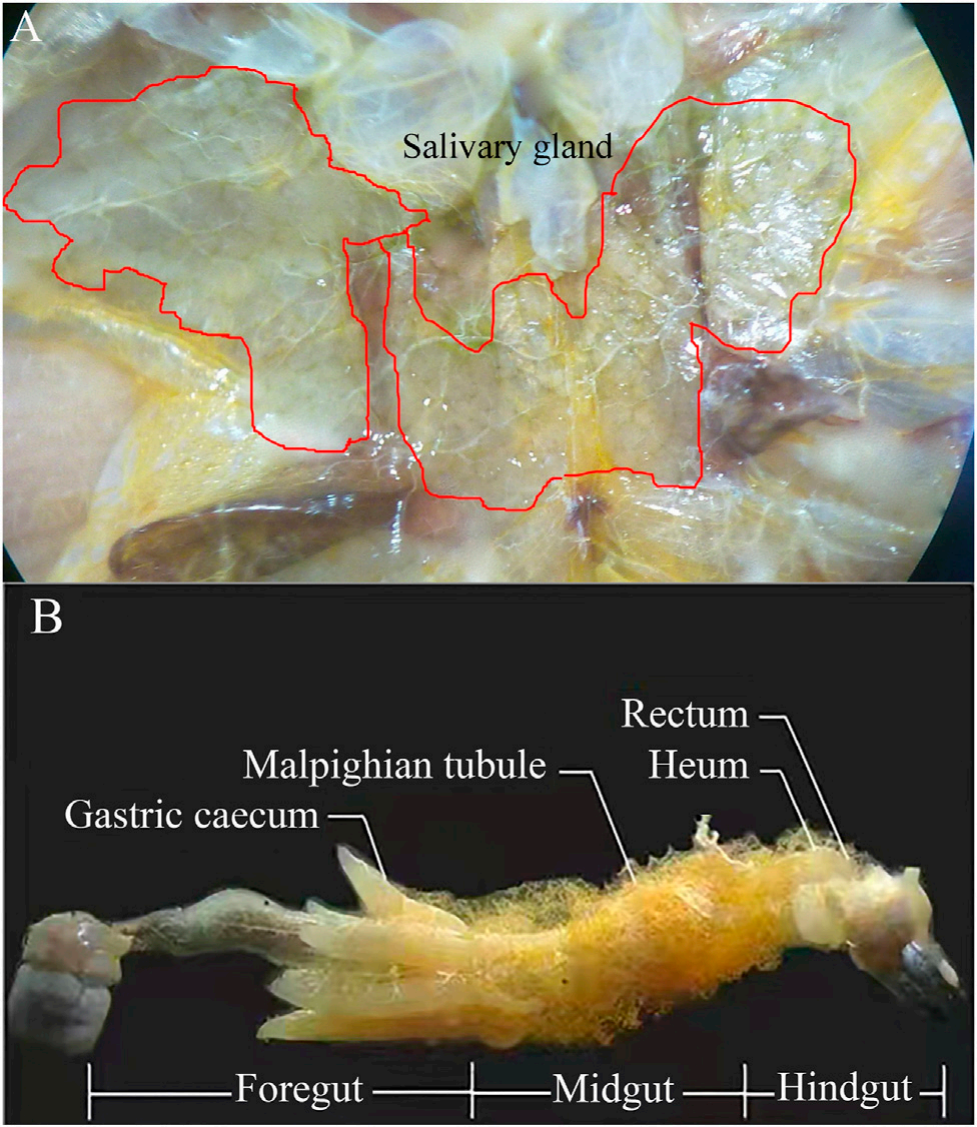
Salivary gland **(A)** and dissection of adult locust digestive tract with labeled regions **(B)**.

The salivary glands, foregut, midgut, and hindgut of the locusts (Figure 1) were dissected on ice using a dissecting needle and ophthalmic forceps. The salivary glands and intestinal contents (foregut, midgut, and hindgut) from 20 adult individuals were taken out, and one part of intestinal contents was soaked in phosphate-buffered saline (PBS) solution (8-g NaCl, 0.2-g KCl, 1.44-g Na_2_HPO_4_, and 0.24-g KH_2_PO_4_, with 1-L deionized water, pH 7.4) and was used to determine the enzymatic activity. The other parts of intestinal contents, feces, orts, and the fresh corn leaves control were ball-milled individually under nitrogen to a fine powder with a Fritsch Planetary Mill (Pulverisette, Germany) using agate bowls and balls for the preservation of the lignin primary structure. Afterward, the fine powders were subjected to further analysis (lignocellulosic content, FE-SEM, FTIR spectroscopy, XRD, and TG analysis/DTG).

### Activity Assays of Carboxymethyl Cellulase, Filter Paper Cellulose, Xylanase, and Manganese Peroxidase

Intestinal contents and salivary glands were fully minced using Micro-Tip Scissors in PBS buffer solution, underwent ultrasonic treatment (ultrasonic intensity 300 W, every ultrasonic time 3 s and interval 4 s, 90 times), and centrifuged at 250 g for 30 min. The supernatant was stored at −80°C as coarse enzyme liquid. CMCase, filter paper cellulase (FPA), and xylanase activities were evaluated by measuring the amount of reducing sugars released from the substrate with a modified 3,5-dinitrosalicylic acid assay (Su et al., 2013). The different substrates were carboxymethyl cellulose (CMC) sodium salt, FP (2 × 3 cm), and xylose separately. One unit of enzymatic activity was defined as the amount of enzyme released from 1 μmol of reducing sugar (glucose or xylose equivalents) per minute (U/mg). Manganese peroxidase (MnPase) activity was determined spectrophotometrically at 470 nm using a Pharma-Spec UV-1700 spectrophotometer (Shimadzu, Japan) from samples taken from the enzyme solution. For the determination of MnPase activity, 2,6-DMP was used as a substrate together with MnSO_4_ and H_2_O_2_ according to a previous report (Wariishi et al., 1992). The amount of enzyme that catalyzes 1-μmol substrate in 1 min was referred to as an enzyme activity unit (U/mg). In this study, all the experiments were conducted in triplicate, and the statistical analysis of the results was performed using Origin 2018 software.

### Cellulose, Hemicellulose, and Lignin Content Analysis

To determine the cellulose, hemicellulose, and lignin content of the dry matter of intestinal contents and feces in locusts, a concentrated sulfuric acid hydrolysis method was used according to a previous report (Sluiter et al., 2012) with minor modifications as described below. The samples were successively extracted in a Soxhlet apparatus using ethanol (200 ml/g) to remove resin and pigment; afterward, a two-step hydrolysis with 72% concentrated sulfuric acid and 4% dilute sulfuric acid to hydrolyze cellulose and hemicellulose into glucose and xylose, respectively, was carried out. Then, the concentrations of glucose and xylose were measured using high-performance liquid chromatography (LC-20 AD, refractive index detector RID-10A, Shimadzu, Kyoto, Japan) with the Aminex HPX-87H column (Bio-rad, Hercules, United States) at 65°C using the mobile phase of 5 mM H_2_SO_4_ at a flow rate of 0.6 ml/min. The lignin content of extractive-free samples was estimated following the method described previously.

### Analysis of Physical and Chemical Structure in Locust Feces

FE-SEM analysis: The surface ultrastructure of samples was observed by using a JSM-7001F field emission scanning electron microscope (JEOL, Japan) after conductive treatment. The corn leaves and locust feces were cut into fragments of approximately 4 mm^2^. The fragments were immersed in a fixative solution (5% formaldehyde, 90% ethanol, and 5% acetic acid) for 24 h at room temperature and then were dehydrated with an increasing series of ethanol (70%, 80%, 90%, and 100%). The samples were glued on “stubs” with the adaxial and abaxial surfaces facing up and covered with carbon. The locust feces and fresh corn leaves were analyzed by FE-SEM to correlate directly with the modification resulting from the digestion process of the locusts.

FTIR spectroscopy assays: FTIR provided information on the lignocellulosic structural changes of corn leaves involving the functional groups after going through the gut of the locust. After the samples were ground into powder in a mortar and with KBr tablet treatment, the transmissivity was determined using a Nicolet 5700 FTIR spectroscope (TMAG, United States) using 2 mg of each sample and scanning ranges from 400 to 4,000 cm^−1^. Baseline and attenuated total reflection corrections for penetration depth and frequency variations were applied using the Shimadzu IR solution 1.30 software supplied with the equipment.

XRD: The crystallinity of corn leaves and locust feces was compared using the XRD profiles. The D8-ADVANCE X-ray diffractometer (Bruker, Germany) was used with the following settings: the X-ray source was copper target, 1.5406 Å wavelength, 40 Kv pipe pressure, DS slit 1, RS 0.2 mm slit, SS 0.2 mm slit with a scanning rate of 4°, and sampling over 0.04 interval time. The relative crystallinity of treatment materials was obtained using the Segal formula (Inoue et al., 2008).

TG analysis: TG analysis is based on the precise study of weight loss during programmed exposure to temperature, to determine digesting-induced changes in the general characteristics of lignocellulose decomposition and activation energies for bond cleavage under pyrolysis and combustion. The determination conditions of a DSC-60 differential scanning calorimeter (TA, United States) were that approximately 5-mg samples were loaded individually in an aluminum pan and vaporized under a nitrogen atmosphere with a flow rate of 20 ml/min and the heating rate was 10°C/min. The TG curve of samples could be measured accurately.

## Results and Discussions

### Lignocellulolytic Enzyme Activity of Locust

In order to understand the spatial expression of lignocellulolytic enzyme in the different segments of the digestive tract in locusts, the lignocellulolytic enzyme of the locust gut contents within salivary glands and the foregut, midgut, and hindgut segments (Figure 1) was evaluated using FP, CMC, xylose, and 2,6-DMP as substrates.

**FIGURE 2:**
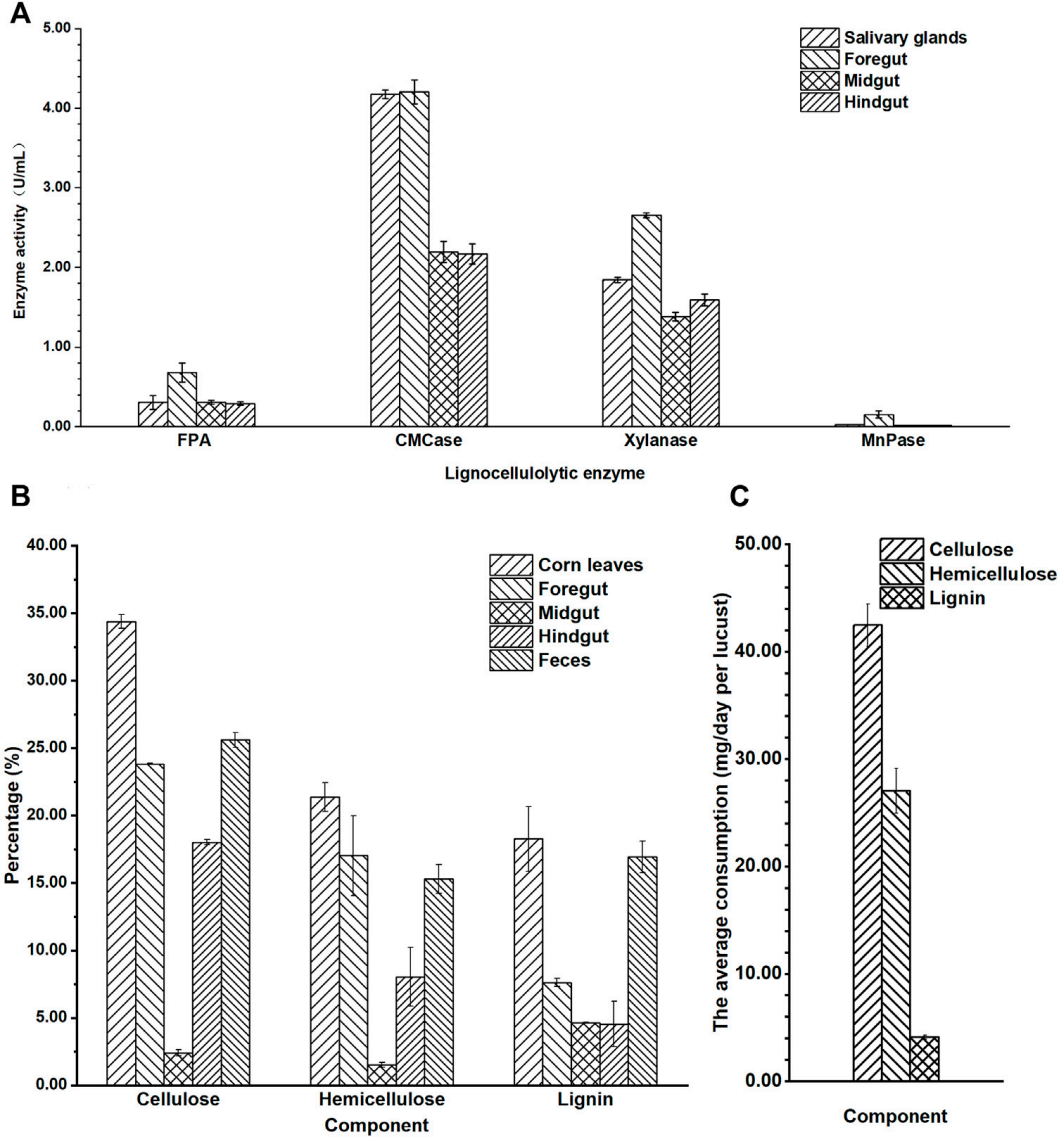
The lignocellulosic degradation of locust. **(A)** Quantitative determination of filter paper cellulase (FPA), carboxymethyl cellulase (CMCase), xylanase, and Mn peroxidase (MnPase) activities in the gut regions of the adult locust. **(B)** The content of cellulose, hemicellulose, and lignin in the different parts of the digestive system in the locust. **(C)** The average consumption of lignocellulose per locust in 1 day.

The activities of FPA, CMCase, xylanase, and MnPase showed similar tendency: foregut > salivary glands > midgut > hindgut (Figure 2A). The highest lignocellulolytic enzyme activities were localized in the foregut segment (FPA 0.68 U/mg, CMCase 4.21 U/mg, xylanase 2.66 U/mg, and MnPase 0.15 U/mg, separately), while they were significantly reduced in the midgut and hindgut regions (*p* ≤ 0.01). The activities of FPA, CMCase, xylanase, and MnPase in the foregut were 2.3, 1.9, 1.7, and 7.7 times the relative activities in the hindgut separately. The activities of FPA, xylanase, and MnPase were revealed to be higher in the foregut than in salivary glands, while the CMCase activity was similar in the foregut and salivary glands, which verified that both of them have a stronger secretory ability of CMCase. No significant differences were detected when comparing the activities of FPA, CMCase, and MnPase in the midgut and hindgut, expect for xylanase. It could be speculated from these results that the major segment of lignocellulose degradation in locusts was the foregut, followed by the midgut and hindgut.

The average activity of CMCase (3.18 U/mg) in the gut was significantly higher than that of xylanase (1.86 U/mg), and MnPase activity (0.05 U/mg) was lowest (*p* ≤ 0.01). These results showed that the basic nutritional components of locusts from corn leaves were cellulose and hemicellulose, not lignin.

### Lignocellulosic Degradation of Locust

To further explain the special characteristic of degradation in the digestive tract, the content of cellulose, hemicellulose, and lignin in the different segments of the gut and feces after the locusts were fed corn leaves for 1 week was analyzed (Figure 2B). The content of the three lignocellulosic components (cellulose, hemicellulose, and lignin) was mostly in corn leaves, and then in the feces, foregut, hindgut, and midgut. The lignocellulosic content descended gradually from corn leaves to the foregut, with the lowest content in the midgut, and then a gradual increasing trend was seen from the midgut to the hindgut and feces. The content of cellulose, hemicellulose, and lignin in the midgut was the lowest, being 2.41%, 1.50%, and 8.64%, respectively, and they reduced to 31.97%, 19.87%, and 9.62%, respectively, lower than those of corn leaves (cellulose 34.38%, hemicellulose 21.37%, and lignin 18.26%). In the hindgut, the content of cellulose and hemicellulose suddenly increased accumulation compared with that in the midgut (being 7.47 and 5.36 times, respectively), while there was no significant difference in lignin. The content of lignin decreased gradually from the foregut, to the midgut, and to the hindgut compared with that of corn leaves, and then suddenly it increased accumulation in feces (approximately 2.6 times).

From the consumption of each locust in a day (Figure 2C), cellulose was the major component digested and absorbed in locusts (42.4 mg), followed by hemicellulose (27.0 mg) and lignin (4.1 mg), with a significant difference (*p* ≤ 0.01).

### The Analysis of the Field Emission Scanning Electron Microscopy of Corn Leaves and Locust Feces

**FIGURE 3:**
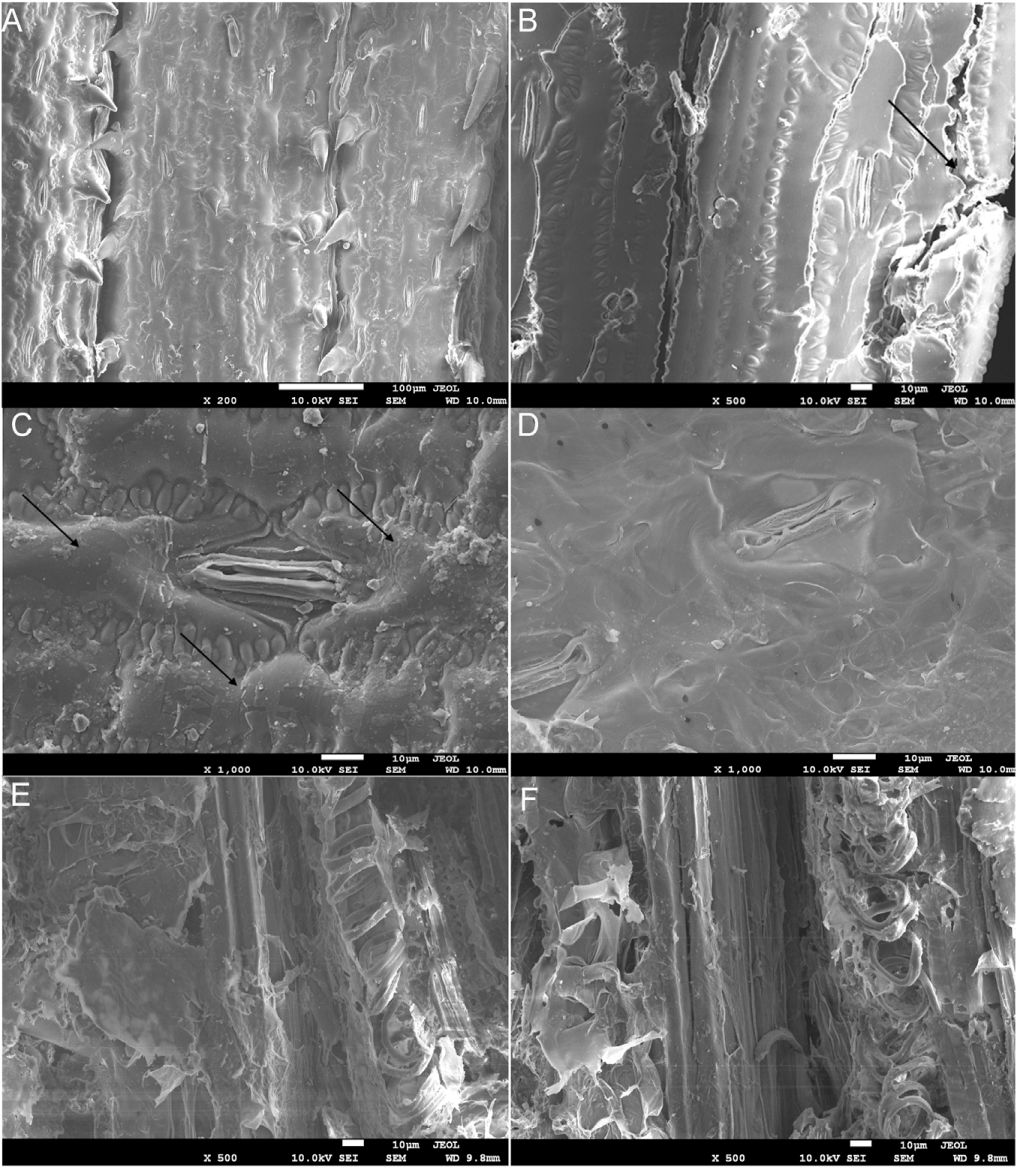
The field emission scanning electron microscopy (FE-SEM) charts of corn leaves **(A)** and undigested corn leaves residue in locust feces **(B–F)**. Note: icon size 10 μm; accelerating voltage 10.0 kV; A–D working distance 10.0 mm, E and F 9.8 mm; amplification of A × 200, B × 500, C and D × 1,000, and E and F × 500. The arrows indicate the edge of the corn leaf being laniated in **(B)**. The arrows indicate silicrete in the cell surface had fallen out using enzymatic hydrolysis in the digestive tract in **(C)**.

The surface structure of corn leaves (Figure 3A) and undigested residue of corn leaves in feces (Figures 3B–F) were analyzed by FE-SEM. After chewing and digesting by the locusts, the surface structure of corn leaves was subjected to different degrees of destruction, and this decomposition got more and more thorough, as shown in Figures 3B–F. Compare with control (Figure 3A), the edge of the corn leaves was laniated, as shown in Figure 3B (indicated by the black arrows), and epidermal hair near the leaf vein had fallen out by mixing or grinding in the digestive tract. Silicrete in the cell surface had fallen out by enzymatic hydrolysis in the digestive tract (indicated by the black arrows in Figure 3C), but the stomatal apparatus and cell wall were kept perfectly (Figure 3C). The stratum corneum of the cell surface had decomposed completely and the cell boundary was out of sight, as shown in Figure 3D. The corn leaves had been decomposed more completely in Figures 3E,F. Mesophyll tissue had been digested and assimilated, and the phloem and thickened part of the xylem in the tube wall of the vascular bundle with high lignin content could be observed clearly. Moreover, the un-thickened part of cellulose and hemicellulose had been broken down.

### Fourier Transform Infrared Spectroscopy Analysis of Corn Leaves and Locust Feces

**FIGURE 4:**
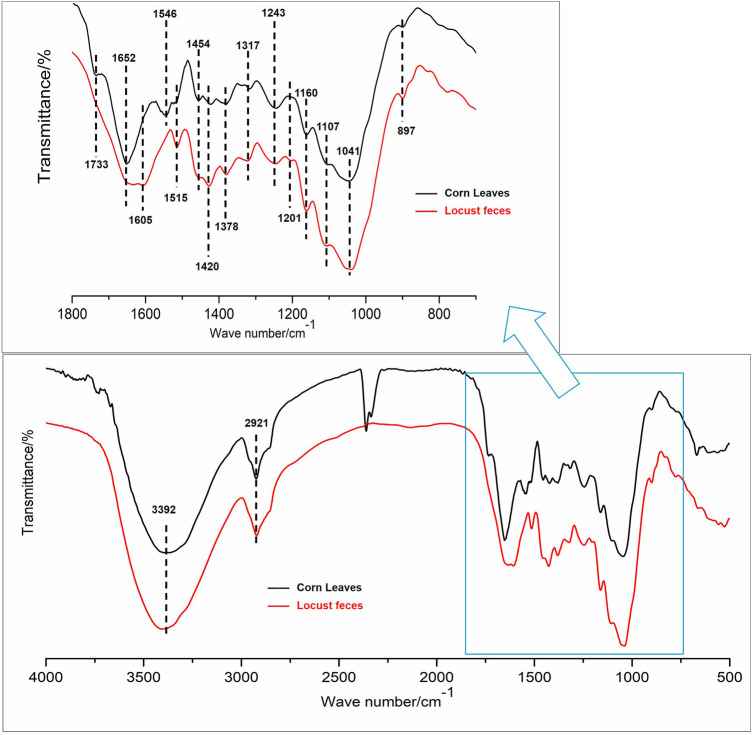
Selected Fourier transform infrared (FTIR) spectroscopy spectra, 400–4,000 cm^−1^ region, for functional group changes by the digestion.

FTIR spectra comparison of corn leaves and locust feces are shown in Figure 4, and the assignments of the functional groups in the two samples are shown in Table 1. From the functional groups related to cellulose and hemicellulose (Song et al., 2005; Ke et al., 2011), the FTIR transmittance of feces significantly increased intensity in peaks 1, 2, 3, 10, 21, 22, and 25. This could be attributed to the metabolism of N–H and –OH on associating with hydroxybenzene and alcohol, the absorption band of methyl, of C–O–C on esters in peaks 1, 2, and 3. Greater intensities of both symmetric bending of aliphatic C–H and C–O stretching in alcohols in peaks 12 and 19 suggested the existence of polysaccharides in the locust feces.

**TABLE 1:** Main assignments of cellulose, hemicellulose, lignin, polysaccharide, and protein in FTIR spectrum band.

No.	Wave numbers (cm^−1^)	Assignments
Cellulose/hemicellulose		
1	3,409	Symmetric vibrations of N–H
2	3,000–3,400	Vibrations of –OH on associated with hydroxybenzene and alcohol
3	2,842–2,940	Absorption band of methyl, methylene, and methine
10	1,448–1,461	Deforming vibrations of C–H on methyl and methylene
21	1,040	Stretching vibrations of C–O–C on esters
22	917–921	Deforming vibrations of –CH_2_ at the end of methylene
25	615–637	Deforming vibrations of C–O–H
Lignin		
4	1714–1725	Stretching of C=O unconjugated to aromatic rings (oxidized sidechains)
5	1,655	Stretching of C=O conjugated to aromatic rings
6	1,594–1,609	Aromatic ring vibrations and C=O stretching
7	1,502–1,536	Aromatic ring absorption band
8	1,462–1,464	Asymmetric C–H bending (in CH_3_ and –CH_2_–)
11	1,421–1,424	Aromatic ring vibrations
13	1,365	Symmetric deformation of C–H in methyl groups
14	1,360	Phenolic hydroxyl vibrations
15	1,270	Vibrations of guaiacyl rings and stretching vibrations of C–O bonds
16	1,221–1,240	Aromatic ring absorption band
17	1,216–1,225	C–C, C–O, and C=O stretching (G condensed > G etherified)
18	1,160	Deformation vibrations of C–H bonds on benzene rings
20	1,075–1,090	Deformation vibrations of C–O bonds in secondary alcohols and aliphatic ethers
24	830	Deformation vibrations of C–H bonds on aromatic rings
Polysaccharide		
12	1,370	Symmetric bending of aliphatic C–H
19	1,030–1,170	C–O stretching in alcohols
23	890	β-Glycosidic linkages in pyranose units
Protein		
9	1,516	C=O stretching in amides

From the functional groups related to lignin, the FTIR transmittance of feces significantly increased intensity in peaks 4–8, 11, 13–18, and 20, which was absorption, deforming, or stretching vibrations of aromatic rings, phenolic hydroxyl, guaiacyl rings, or benzene rings, and so on (Song et al., 2005; Ke et al., 2011). The increasing intensity of peaks 8 and 13 of the feces sample can be attributed to the metabolism of the –CH_3_ and –CH_2_ groups. The most obvious change was observed in the range of 1,655 cm^−1^, indicating the greater exposure of the stretching of C=O conjugated to aromatic rings (peak 12).

### X-Ray Diffraction Analysis and Kinetic Thermogravimetric Analysis of Corn Leaves and Locust Feces

**FIGURE 5:**
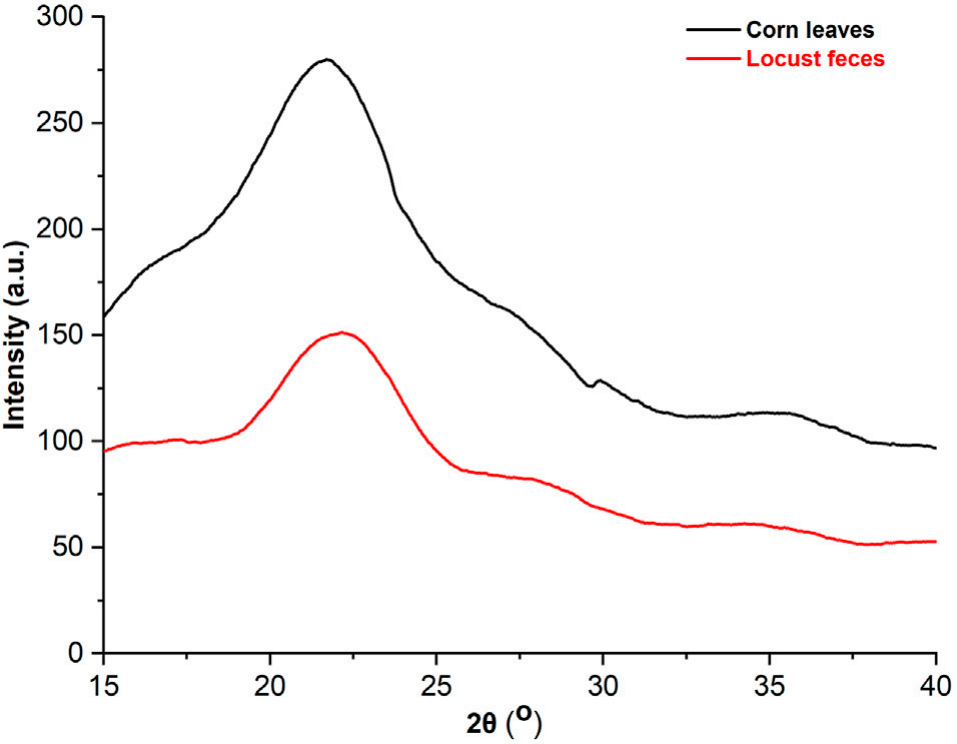
The analysis of the X-ray diffraction (XRD) pattern of corn leaves and locust feces. XRD diffraction conditions: copper target, 1.5406 Å wavelength, 40 Kv pipe pressure, DS slit 1°, RS 0.2-mm slit, SS 0.2-mm slit with a scanning rate of 4°, and sampling over 0.04 interval time.

XRD was applied to analyze the changes in the crystalline and amorphous regions of cellulose, which showed that the characteristic peak of cellulose I at 2θ = 22.3^o^ was observed in both corn leaves and locust feces, and the spectra are presented in Figure 5. The relative crystallinity of corn leaves and locust feces were 30.95% and 37.99%, respectively, which was obtained using the Segal formula. Relative crystallinity in locust feces was increased by 23.4% compared with that in corn leaves, indicating that the corn leaves were damaged after digestion.

**FIGURE 6:**
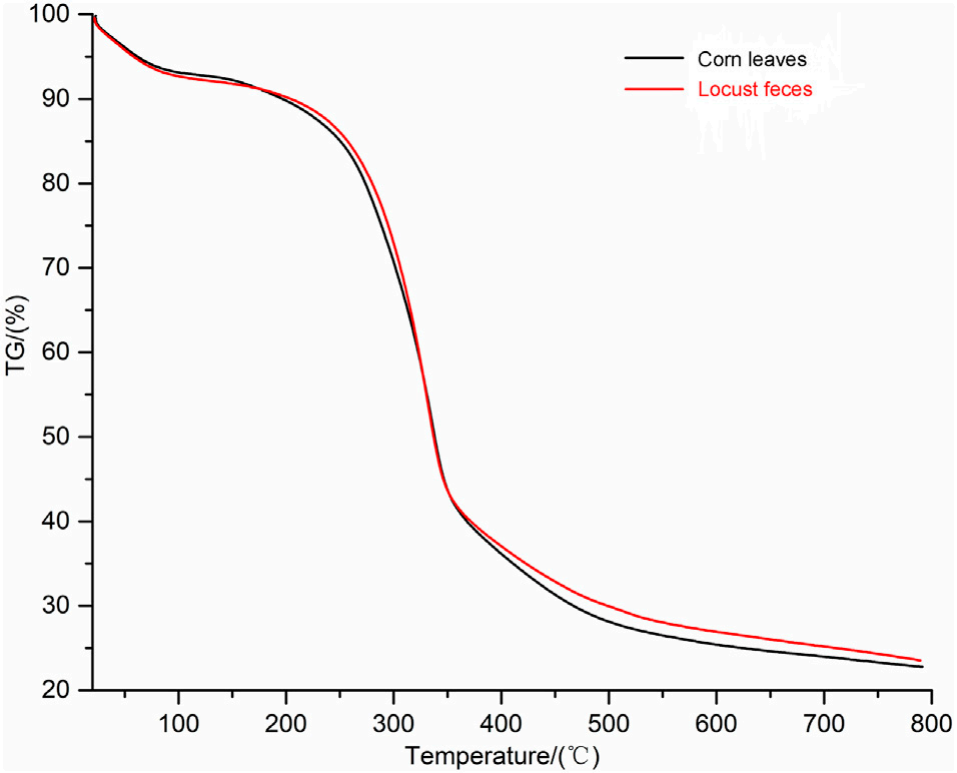
Thermogravimetric (TG) analysis spectra of corn leaves and locust feces. TG analysis kinetics parameters: the heating rate was 10°C/min, and the flow rate of N_2_ was 20 ml/min.

TG analysis was put into effect to investigate the thermal degradation kinetics of both digested (locust feces) and undigested corn leaves samples. In this work, the major chemical components of corn leaves (cellulose, hemicelluloses, and lignin) were degraded at different temperatures. The TG curves of corn leaves and locust feces are shown in Figure 6. TG analysis measured the weight loss as a function of temperature. According to TG analysis, there were two obvious differences in pyrolysis characteristics of corn leaves and locust feces: 1) the volatile content in feces was higher than that of corn leaves, and 2) the temperature interval of locust feces was volatile and tended to be on the high-temperature side compared with that of corn leaves. The volatile content of locust feces reached 76.5%, which was 8.97% higher than that of corn leaves. These results showed that the content of fixed carbon was decreased after digestion and absorption of the locust gut. Because the biggest contributor to fixed carbon content was lignin in lignocellulose fractions, it could be inferred that the lignin content of locust feces was lower than that of corn leaves. The temperature of begin and end to separate out in volatile of locust feces were 289°C and 363°C, which were 22°C and 15°C higher than that of corn leaves respectively. The above results clearly indicate that corn leaves were decomposed or used after the intestinal digestion of locusts.

## Discussions

In nature, different lignocellulose degradation processes exist that rely on the combined action of many lignocellulolytic enzymes present in the digestive tract of phytophagous and xylophagous organisms (Godon et al., 2013; Gales et al., 2018). To date, most studies focused on phytophagous and xylophagous insects, which have been well known as the most effective lignocellulose digesters (Rizzi et al., 2013; Jang and Kikuchi, 2020). However, only few studies have focused on the mechanism of lignocellulose deconstruction in the gut system of herbivorous locusts in recent years. In this study, we examined the enzyme activity related to the degradation of lignocellulose and the content of cellulose, hemicelluloses, and lignin in the different gut segments of *L. migratoria manilensis* after being fed with corn leaves. Meanwhile, a series of characterization analyses were conducted on corn leaves and locust feces. These results provided evidence of lignocellulose structural alterations during such digestion process of gramineous lignocellulose.

As previously observed for alternative insect samples, the lignocellulolytic enzymes were localized mainly in the foregut or midgut regions, while greatly reduced activity was detected in the hindgut region. For example, the lignocellulolytic enzymes were localized to the midgut regions of the *D. carolina* digestive tract, and then database searches indicated high similarity with the endo-β-1,4-glucanases from invertebrates, bacteria, and plants (Willis et al., 2010). The relative expression levels of 12 digestive enzyme genes in the midgut were significantly higher than those in the other tissues for *Eucryptorrhynchus scrobiculatus* (Gao et al., 2020). High endo-β-1,4-glucanase activity was detected in the midgut of the *Eurycantha calcarata* (Shelomi et al., 2014). In this study, locusts were fed corn leaves for 1 week, and the majority of lignocellulolytic enzyme activities were localized to the salivary glands and foregut regions, while they significantly reduced in the midgut and hindgut regions, which indicated that the main spatial part of lignocellulose degradation was in the foregut of locusts. This conclusion indicated that the location of lignocellulolytic enzyme enrichment in locusts was a little different from the above-mentioned insects. The activities of FPA, CMCase, and xylanase in the foregut were higher than those in the midgut and hindgut (Figure 2A), which is probably due to the salivary glands of locusts including many released digestive enzymes that would flow into the foregut and catalyze the hydrolysis of the cellulose and hemicellulose in the foregut region. In this study, the highest activity of CMCase was in the salivary glands and foregut but not in the midgut, which was somewhat different from previous studies.

From the content of cellulose, hemicellulose, and lignin in the different parts of the gut and feces, it was speculated that the locusts had strong lignocellulosic degradation ability in this study. The lower content of cellulose, hemicellulose, and lignin in the midgut than in the foregut indicated that the main region of absorption should be the midgut, while the rest of the unabsorbed ingredients in the midgut would be concentrated in the hindgut, leading to the content of corn leaves in the hindgut and feces being increased significantly. Quantitative and qualitative assays of cellulase identified the foregut as the region with the highest levels of cellulase activity in both *Thermobia domestica* and *Ctenolepisma longicaudata* (Pothula et al., 2019). The midgut had also been identified as the main biological treatment region; alkaline conditions of the midgut could enhance the dissolution of lignin and deesterification of intermolecular ester bonds (Gales et al., 2018), which could further increase the surface area and porosity and decrease the crystallinity of the biomass (Kim et al., 2016; Ozbayram et al., 2020). Hence, combining the data on the enzyme activity and content of lignocellulose, it could be reasonable to infer that the major decomposition of lignocellulose was accomplished in the foregut and midgut and the majority of nutritional components were absorbed in the midgut of locusts. According to the nutrient consumption of each locust in a day, the major component digested and absorbed by the locusts was cellulose, followed by hemicellulose. Meanwhile, only 4.1-mg lignin (1/10th of cellulose) was consumed. If lignin was degraded largely by locusts, many inhibitors would accumulate, which would not be conducive to the degradation of cellulose (Huang et al., 2022). However, there are some interesting results that need to be explored further. One is that even if the hemicellulose component were degraded into sugar monomers, the locusts could not utilize pentoses, such as xylose and arabinose (Dadd, 1960), which constituted the majority of hemicellulose polysaccharides in gramineous plants (Wilkie, 1979). These pentoses might be utilized by microorganisms in the locust guts.

According to the results of FE-SEM, the surface structure of corn leaves was subjected to different degrees of destruction after mastication and digestion. It showed that the lignocellulose of corn leaves could be digested, assimilated, and utilized by the locusts, especially cellulose and hemicellulose. The cell wall degrading enzymes (such as CMCase) could increase the pore size of the pit membrane, and separate adjacent xylem vessels (Pérez-Donoso et al., 2010). Nevertheless, the xylem in the tube wall of the vascular bundle resists lignocellulose degradation in locusts; as shown by some experimental evidence, the bundle sheath cells appear to pass through the digestive system intact (Caswell and Reed, 1976). Therefore, for orthopteran herbivores, the lignin contents might be considered a barrier that locusts must mechanically rupture to assimilate nutrients and may be more important than was previously thought. It would be contributing as a diluent of the more easily digested cell contents (Clissold et al., 2004). In Figure 3, the surface structure of locust feces became rough. A similar phenomenon occurred with bamboo after conventional acid pretreatment (Lin et al., 2021), which suggested that the biodegradation of the locust gut could also achieve the effect of traditional acid pretreatment.

FTIR spectra comparison of corn leaves and locust feces could analyze the changes in the functional groups during the degradation of corn leaves. In this study, the FTIR transmittance of feces increased significantly, and intensity in the functional groups represented cellulose and hemicellulose, which was because the content of cellulose and hemicellulose in feces decreased significantly. It indicated that the cellulose and hemicellulose had been degraded to glucose and xylose, absorbed, and utilized by the locust gut. In addition, according to the functional groups related to lignin, the most obvious change was observed in the range of 1,655 cm^−1^. It indicated that a greater exposure emerged by the stretching of C=O conjugated to aromatic rings (peak 12). It could be speculated that the removal of C=O conjugated to the aromatic ring group will help alleviate the steric hindrance effect of lignin to enzymes and may help the downstream hydrolysis of cellulose and hemicellulose (Ke et al., 2012). These results indicated that the lignin side-chain structure of corn leaves was modified after digestion and absorption of locusts. In short, the FTIR results of locust feces showed that the intestinal tract of locusts could degrade the corn leaves in two ways: 1) the functional groups of cellulose and hemicellulose (such as N–H, –OH, C–H, C–O–C, and –CH_2_) had been degraded, absorbed, and utilized by the locust gut and 2) the functional groups of lignin (such as C=O and C–H aromatic rings, phenolic hydroxyl, guaiacyl rings, C–C, and C–O) had been dissociated and performed a side-chain modification of lignin as well as rearrangement of the modified lignin fragments by the locust gut. Furthermore, the majority of the lignin modifications occurred on G lignin sites; meanwhile, G lignin sites had scarcely any modifications in the lignin polymeric framework during the digestion by the locust gut.

After XRD, the relative crystallinity of cellulose in locust feces was significantly increased compared with that in corn leaves. The reason may be that the surface areas of amorphous and crystalline regions overflowed in corn leaves and the crystalline region was exposed outside after digestion and absorption of locusts. Some researchers found that the relative crystallinity of cellulose in sorghum straw increased from 48.7% to 65.5% after weak acid pretreatment (Dai et al., 2022). These results indicated that the biodegradation of locusts also had a similar effect compared with the conventional pretreatment.

The TG method was a technique to measure the relationship between mass and temperature under programmed temperature control. In this study, the major chemical components of corn leaves (cellulose, hemicelluloses, and lignin) degraded at different temperatures. The highly crystalline cellulose was thermally stable (Sharypov et al., 2002), and the amorphous hemicelluloses and lignin started decomposition before cellulose did (Hill, 2006), with hemicelluloses being the least thermally stable components on account of the acetyl groups (Ke et al., 2011), at approximately 300°C. Furthermore, lignin starts to degrade at relatively low temperatures, over a wide temperature range, at approximately 230°C. In this study, the data of TG analysis were consistent with the results of other previous characterization analyses, which ensured the accuracy of the results.

## Conclusion

In this study, the locust was chosen as the research object and was fed fresh corn leaves for 1 week. In this process, the lignocellulolytic enzyme activities and the lignocellulosic contents in the different parts of the digestive tract were analyzed first. Then, the characterizations of lignocellulosic degradation after digestion in the digestive tract of the locusts were represented comprehensively using FE-SEM, XRD, FTIR, and TG analysis. The data proved conclusively the presence of lignocellulolytic enzyme activity and the efficient lignocellulose degradation ability in the digestive system of the locusts. The major decomposition of lignocellulose was accomplished in the foregut and the minor decomposition in the midgut. Nevertheless, the majority of nutritional components were absorbed in the midgut. According to the nutrient consumption of each locust in a day, the major component digested and absorbed by the locusts was cellulose, followed by hemicellulose, while the lignin component was barely digested by the locusts. The unique gramineous lignocellulose biodegradation system of insects could contribute to the development of new biorefinery pathways.

## Data Availability

The raw data supporting the conclusions of this article will be made available by the authors, without undue reservation.
